# Plant Extract Synthesized PLA Nanoparticles for Controlled and Sustained Release of Quercetin: A Green Approach

**DOI:** 10.1371/journal.pone.0041230

**Published:** 2012-07-23

**Authors:** Avnesh Kumari, Vineet Kumar, Sudesh Kumar Yadav

**Affiliations:** Biotechnology Division, Council of Scientific and Industrial Research-Institute of Himalayan Bioresource Technology, Council of Scientific and Industrial Research, Palampur (HP), India; Universidad de Castilla-La Mancha, Spain

## Abstract

**Background:**

Green synthesis of metallic nanoparticles (NPs) has been extensively carried out by using plant extracts (PEs) which have property of stabilizers/ emulsifiers. To our knowledge, there is no comprehensive study on applying a green approach using PEs for fabrication of biodegradable PLA NPs. Conventional methods rely on molecules like polyvinyl alcohol, polyethylene glycol, D-alpha-tocopheryl poly(ethylene glycol 1000) succinate as stabilizers**/**emulsifiers for the synthesis of such biodegradable NPs which are known to be toxic. So, there is urgent need to look for stabilizers which are biogenic and non-toxic. The present study investigated use of PEs as stabilizers/emulsifiers for the fabrication of stable PLA NPs. Synthesized PLA NPs through this green process were explored for controlled release of the well known antioxidant molecule quercetin.

**Methodology/Principal Findings:**

Stable PLA NPs were synthesized using leaf extracts of medicinally important plants like *Syzygium cumini* (1), *Bauhinia variegata* (2), *Cedrus deodara* (3), *Lonicera japonica* (4) and *Eleaocarpus sphaericus* (5). Small and uniformly distributed NPs in the size range 70±30 nm to 143±36 nm were formed with these PEs. To explore such NPs for drugs/ small molecules delivery, we have successfully encapsulated quercetin a lipophilic molecule on a most uniformly distributed PLA-4 NPs synthesized using *Lonicera japonica* leaf extract. Quercetin loaded PLA-4 NPs were observed for slow and sustained release of quercetin molecule.

**Conclusions:**

This green approach based on PEs mediated synthesis of stable PLA NPs pave the way for encapsulating drug/small molecules, nutraceuticals and other bioactive ingredients for safer cellular uptake, biodistribution and targeted delivery. Hence, such PEs synthesized PLA NPs would be useful to enhance the therapeutic efficacy of encapsulated small molecules/drugs. Furthermore, different types of plants can be explored for the synthesis of PLA as well as other polymeric NPs of smaller size.

## Introduction

Green nanotechnology involves the application of green chemistry principles for the fabrication of nanoscale materials [1], [2]. Green chemistry strives to discover synthetic methods that eliminate harmful reagents and enhance the efficiency of existing methods. Due to these reasons green chemistry approach is preferred nowadays for the synthesis of nanomaterials [3], [4]. Such nanomaterials would be no/less harmful to human beings.

Green synthesis of metallic NPs by using plant extracts (PEs) have been extensively investigated [5], [6], [7], [8], [9], [10], [11], [12], [13]. PEs contain valuable compounds like saponins, terpenoids, proteins, polyphenols and flavonoids. These compounds have the properties of stabilizers/ emulsifiers [14]. However, to best of our knowledge till date use of PEs for fabrication of Poly(D,L-lactide) (PLA) NPs has not been reported. Biodegradable NPs are of importance due to their wide applications in nanomedicines [15], [16]. Among the biodegradable NPs, PLA is extensively investigated for encapsulation and delivery of drugs/small molecules [15]. Preparation of PLA NPs involves the formation of emulsions. Emulsions are unstable without the presence of stabilizers/emulsifiers. Most of synthetic methods rely heavily on surfactants like polyvinyl alcohol (PVA), polyethylene glycol (PEG) and polyvinylpyrrolidone (PVP) [15]. Conventional NPs are rapidly eliminated from reticuloendothelial system within seconds or few minutes [17]. In conventional methods, NPs surface are modified by stabilizers (hydrophilic polymers) like PEG, PVA to increase their persistence in the blood. These stabilizers remain on the surface of NPs even after several washings. Hence their half life increases, which can cause toxicity [18], [19]. So there is urgent need to look for stabilizers which are biogenic and non toxic.

In view of this, we have investigated here the use of PEs as stabilizers/ emulsifiers for the synthesis of stable PLA NPs. Extracts of medicinally important plants *Syzygium cumini*, *Bauhinia variegata*, *Cedrus deodara*, *Lonicera japonica* and *Eleaocarpus sphaericus* were used as stabilizers/emulsifiers for the synthesis of PLA-1, PLA-2, PLA-3, PLA-4 and PLA-5 NPs, respectively. PEs synthesized PLA NPs were characterised by SEM, TEM, Nanodrop and DLS. As PLA NPs prepared by other methods have been widely investigated for small molecules delivery to enhance their stability, solubility and bioavailability, we have tested PE synthesized PLA-4 NPs for controlled and sustained release of the well known antioxidant molecule quercetin (Mol formula: C_15_H_10_O_7_). The chemical structure of quercetin molecule is shown in Fig. S1. Quercetin has been proven to possess pharmacological properties, however use of quercetin in pharmaceutical field is limited due to low aqueous solubility, poor skin permeability, instability and extensive first pass metabolism before reaching the systemic circulation [20], [21], [22]. These problems can be minimised by encapsulating quercetin on PEs synthesized PLA NPs [21], [22] . The encapsulation efficiency, drug loading and *in vitro* release of quercetin from PLA-4 NPs were quantified by validated HPLC method [23].

## Results

### Synthesis of PLA Nanoparticles Using PEs and their Characterization

The five medicinally important plants namely *Syzygium cumini*, *Bauhinia variegata*, *Cedrus deodara*, *Lonicera japonica* and *Eleaocarpus sphaericus* were used as stabilizers/emulsifiers for the synthesis of biodegradable PLA NPs. These plants extracts (PEs) showed very good potential for synthesis of PLA (PLA-1, PLA-2, PLA-3, PLA-4 and PLA-5) NPs. Sonication of PLA solution in DCM and PEs in water as stabilizer solution was resulted in the formation of stable emulsion that ultimately led to synthesis of stable PLA NPs. Control experiment was performed without the PEs to establish the role of PEs in the synthesis of PLA NPs. PLA NPs were not formed in the absence of PEs (Fig. S2).

**Figure 1:**
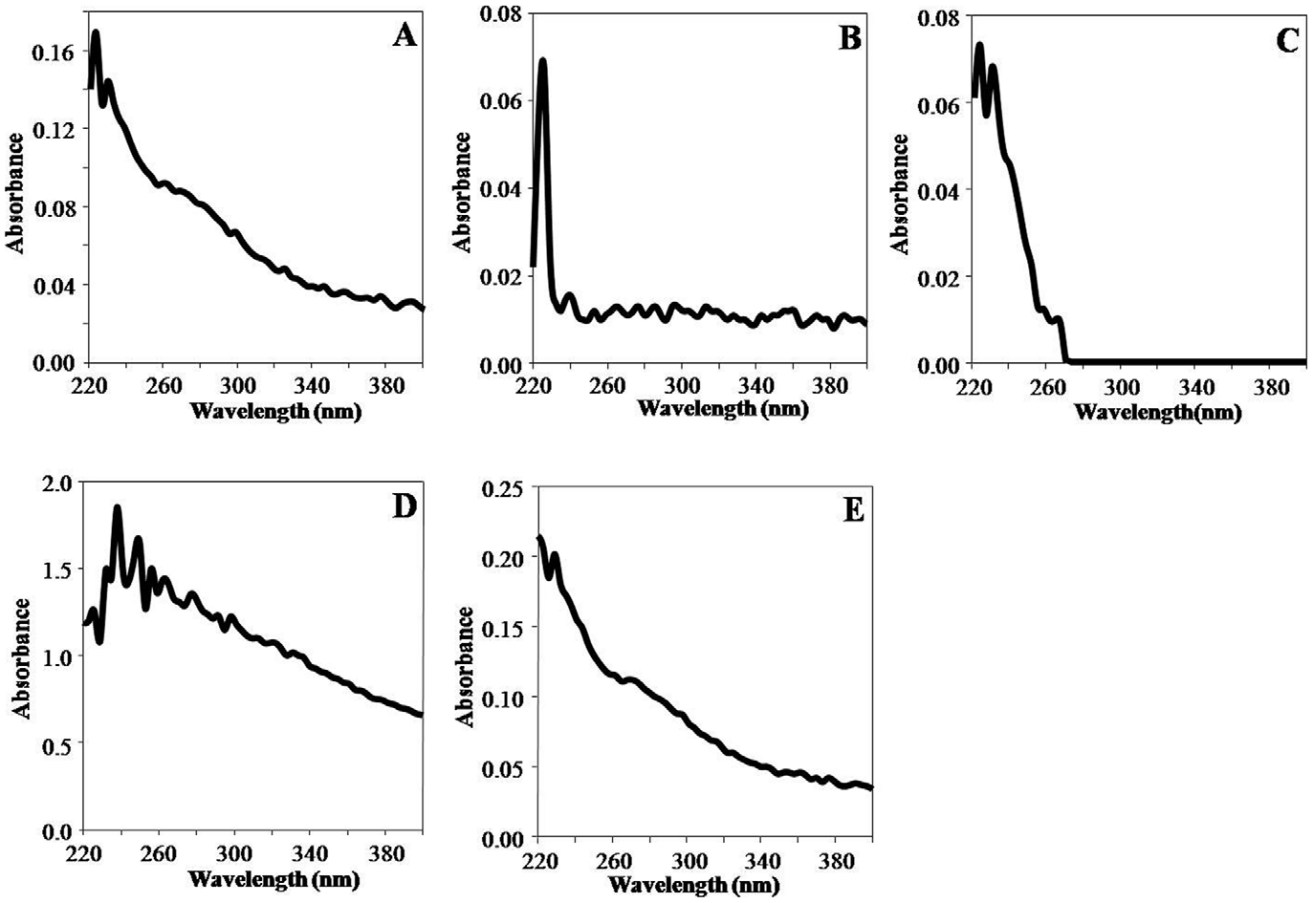
UV-visible spectra of PLA NPs synthesized using PEs as stabilizer/emulsifier. (A) PLA-1 (B) PLA-2 (C) PLA-3 (D) PLA-4 (E) PLA-5. UV-Vis spectra were recorded on nanodrop spectrophotometer by loading 2 µl of each PEs synthesized PLA NPs.

**Figure 2:**
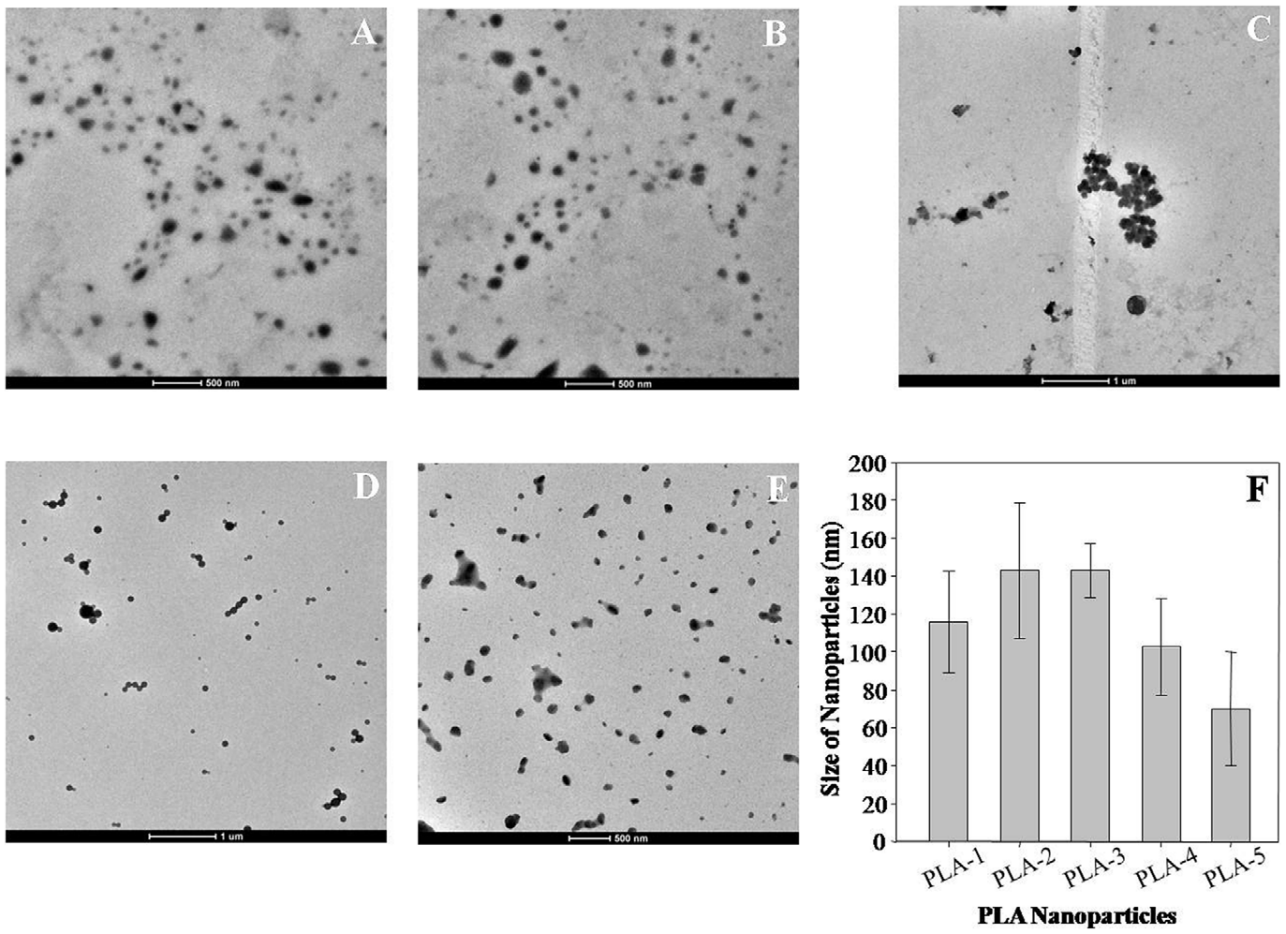
Transmission electron micrographs of PLA NPs synthesized using PEs as stabilizer/emulsifier. (A) PLA-1 (B) PLA-2 (C) PLA-3 (D) PLA-4 (E) PLA-5. A drop of each PEs synthesized PLA NPs were loaded on copper grids, negatively stained with 2% ammonium molybdate and images were recorded on Tecnai T20 twin, TEM at 200 kV. Bar diagram showed the size of various PLA NPs (F).

**Figure 3:**
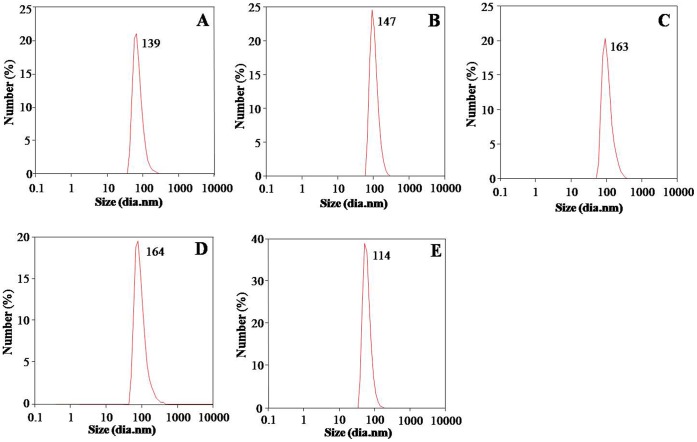
Size measurements of PLA NPs with DLS. (A) PLA-1 (B) PLA-2 (C) PLA-3 (D) PLA-4 (E) PLA-5. One ml of each PEs synthesized PLA NPs was taken in disposable sizing cuvette cells for recording size on DLS.

PEs synthesized PLA NPs were characterised by UV-Spectroscopy, SEM, TEM and DLS. UV-visible absorption spectroscopy was used for screening of reaction mixtures for synthesis of PLA NPs. All five PEs synthesis reaction mixtures (PLA-1, PLA-2, PLA-3, PLA-4 and PLA-5) showed peak near 227 nm i.e. characteristic of PLA NPs [23]. This indicated synthesis of PLA NPs (Fig. 1A–E). SEM was used for initial confirmation of PLA NPs synthesis. SEM analysis revealed synthesis of similar sized, spherical NPs with smooth surface (Fig. S3A–E). TEM was used for exact morphological characterization. TEM characterisation of PE synthesized PLA NPs revealed that all the NPs were stable, well dispersed, smooth and spherical in shape. Different leaf extract directed different sized PLA NPs (Fig. 2A–E). PLA NPs synthesized by *S. cumini*, *B. variegata*, *C. deodara*, *L. japonica* and *E. sphaericus* were 116±27 nm (PLA-1), 143±36 nm (PLA-2), 143±14 nm (PLA-3), 103±26 nm (PLA-4) and 70±30 nm (PLA-5) in size, respectively (Fig. 2F). The results expressed were average of three consecutively synthesized NPs by same method. Dynamic light scattering (DLS) characterisation reveals that PLA NPs synthesized by *S. cumini*, *B. variegata*, *C. deodara*, *L. japonica* and *E. sphaericus* PEs were 139±15 nm (PLA-1) , 147± 10 nm (PLA-2),164±20 nm (PLA-3), 164±10 nm (PLA-4) and 114± 70 nm (PLA-5), respectively (Fig. 3A–E). Polydispersity index (PDI) of PLA NPs synthesized by *S. cumini*, *B. variegata*, *C. deodara*, *L. japonica* and *E. sphaericus* PEs were 0.18 (PLA-1), 0.19 (PLA-2), 0.2 (PLA-3), 0.1 (PLA-4) and 0.39 (PLA-5) respectively. PDI < 0.2 indicates monomodal size distribution of NPs. Higher polydispersity index for PLA-5 indicates different sized NPs in the solution. Inconsistency in the size of NPs measured by TEM and DLS is due to the fact that DLS method gives the hydrodynamic diameter of NPs rather than actual size of NPs [24].

**Figure 4:**
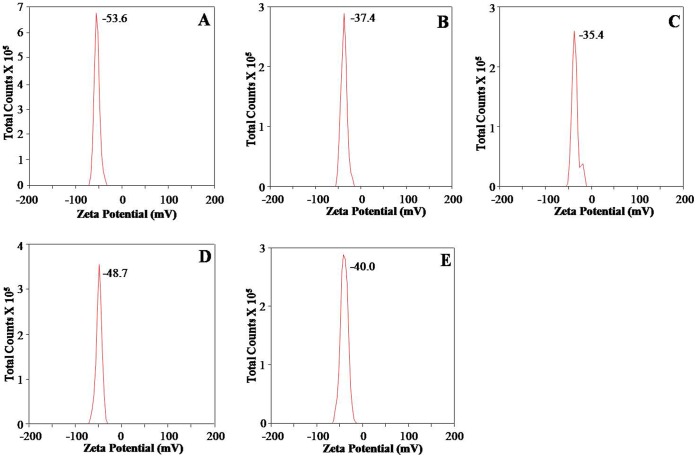
Zeta potential measurements of PLA NPs synthesized using PEs as stabilizer/emulsifier. (A) PLA-1 (B) PLA-2 (C) PLA-3 (D) PLA-4 (E) PLA-5. One ml of each PEs synthesized PLA NPs was taken in disposable zeta cells for recording zeta potential on Zeta sizer Nano ZS. All the NPs showed zeta potential more than −30 mV indicating good stability.

DLS was also used to measure the zeta potential of PEs synthesized PLA NPs. Zeta potential value is indicator of stability of NPs against agglomeration. Zeta potential values of PEs synthesized PLA-1, PLA-2, PLA-3, PLA-4 and PLA-5 NPs were −53.6±0.8, −37.4±0.6, −5.4±1.0, −48.7±1.0 and −40.0±0.8 mV, respectively (Fig. 4A–E). All PEs synthesized PLA NPs showed strong negative zeta potential documenting their stable nature.

### Encapsulation of Quercetin in PLA-4 Nanoparticles and Characterization of Quercetin Loaded PLA-4 Nanoparticles

**Figure 5:**
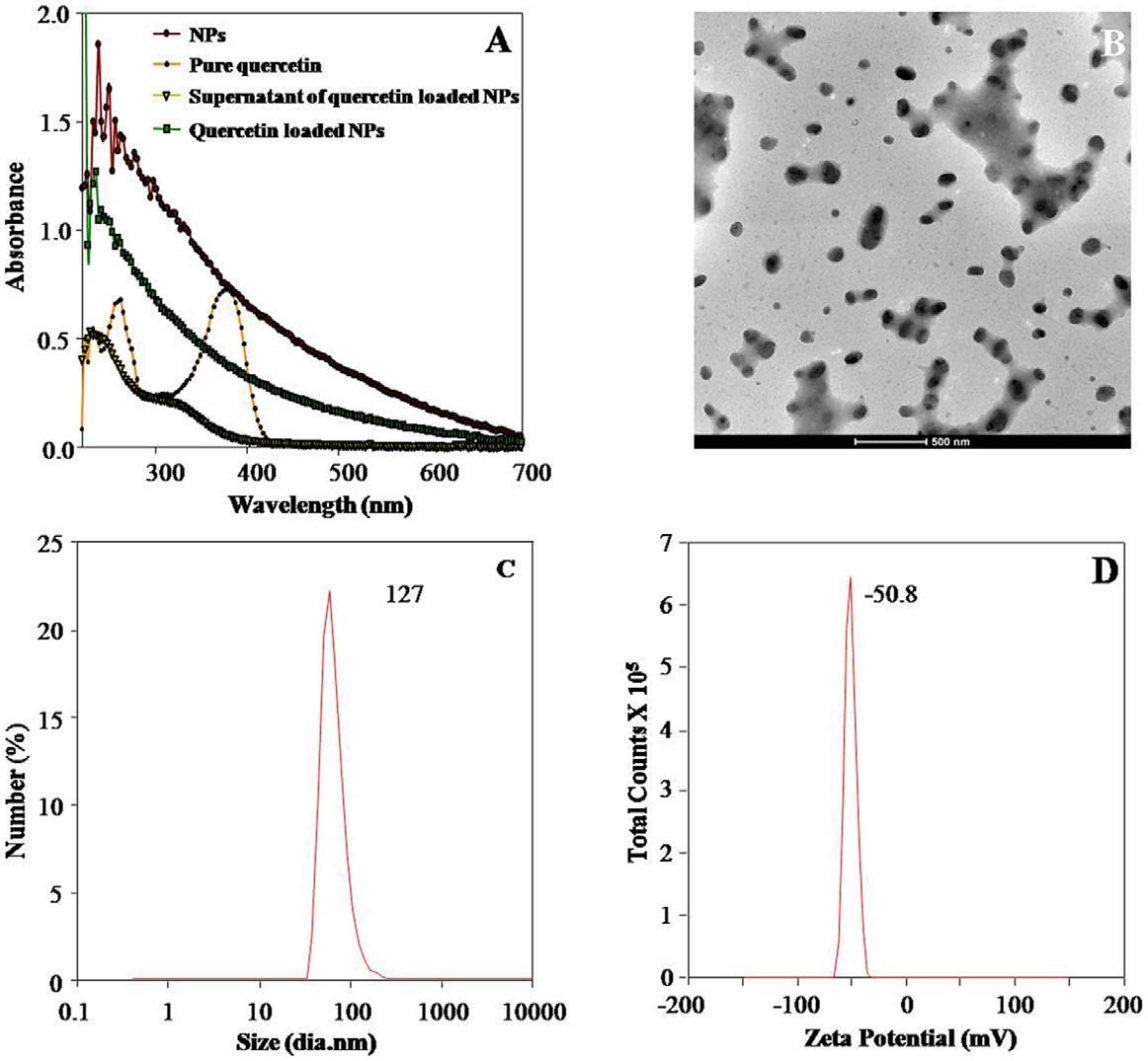
Characterization of quercetin loaded PE synthesized PLA-4 NPs. UV–Vis spectra of free quercetin, PEs synthesized PLA NPs and quercetin loaded PE synthesized PLA-4 NPs (A). Transmission electron micrographs of quercetin loaded PE synthesized PLA-4 NPs (B). DLS size measurements of quercetin loaded PE synthesized PLA-4 NPs (C). Zeta potential of quercetin loaded PE synthesized PLA-4 NPs (D). PLA-4 NPs were more stable and well dispersed.

To check the functionality of PEs synthesized PLA biodegradable NPs, one of the most uniformly distributed PLA-4 NPs established by TEM and DLS, was used to encapsulate the well known antioxidant molecule quercetin. The method used was found to be very effective for encapsulation of quercetin in its fully active form on PLA-4 NPs. Quercetin showed an absorption peak at 350 nm and the intensity of this peak was decreased significantly due to encapsulation of quercetin in PLA-4 NPs. The UV-visible spectra of both PLA-4 NPs and quercetin loaded PLA-4 NPs showed characteristic peaks due to turbidity (Fig. 5A). However, absorbance of quercetin loaded PLA-4 NPs was higher than corresponding blank NPs.

Quercetin encapsulated PLA-4 NPs were 103±35 nm in size (Fig. 5B). There was no significant difference in the size of quercetin loaded PLA-4 NPs as compared to blank PLA-4 NPs (103±26 nm). Zeta potential of quercetin loaded PLA-4 NPs (−50.8±0.6 mV) and blank PLA-4 NPs (−48.7±1.0 mV) suggested equal stability of these NPs (Fig. 5C).

### Encapsulation Efficiency of Quercetin in PLA-4 Nanoparticles Using HPLC

Encapsulation efficiency of quercetin in PE synthesized PLA-4 NPs was quantified using the validated HPLC method [23]. The calibration curve was prepared by analysing different concentrations of free quercetin vs. peak area of eluted peak (Fig. S4A). The concentration of quercetin present in supernatant sample was calculated by regression equation generated by calibration curve (Fig. S4A).
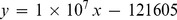
(1)


Where y is the arbitrary area of quercetin eluted peak in HPLC and x is the amount of quercetin in mg/ml. The amount of nanoencapsulated quercetin was back calculated by subtracting the amount of quercetin used in initial formulation from amount of unbound quercetin present in supernatant obtained after centrifugation of quercetin loaded PLA-4 NPs. HPLC analysis revealed that the encapsulation efficiency of quercetin loaded PLA-4 NPs was 100 %, as there was no quercetin peak in the supernatant (Fig. S4B). The actual quercetin loading with respect to mass of the quercetin loaded PLA NPs recovered after lyophilisation as calculated by equation 3 was 13.91 %.

### *In vitro* Release Assay of Quercetin Loaded PLA-4 Nanoparticles

**Figure 6:**
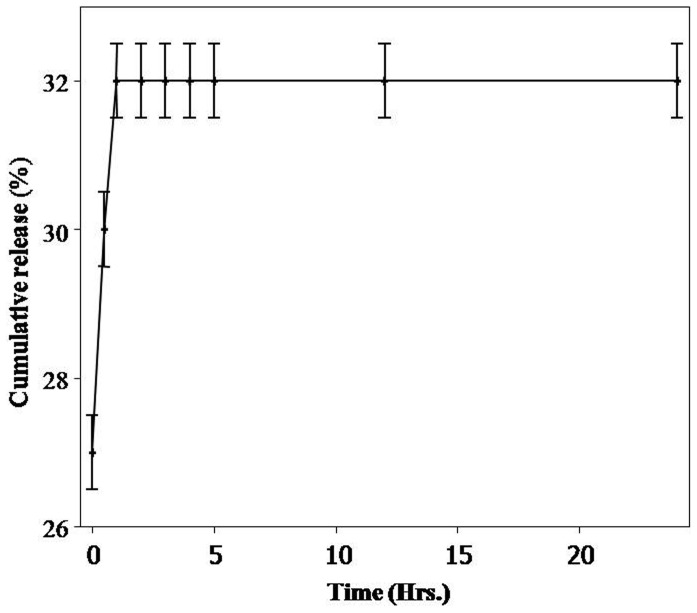
Release profile of quercetin from quercetin loaded PE synthesized PLA-4 NPs by HPLC method. Release curve was obtained by plotting % of quercetin released vs. time.

The *in vitro* release study of quercetin loaded PLA-4 NPs was carried out in phosphate buffer saline to mimic the physiological conditions in the living organisms. Amount of released quercetin was calculated by using standard calibration curve generated by HPLC (Fig. S4A). An average 20–27 % quercetin was released within 0 – 0.5 h showing rapid burst release. The maximum release of quercetin was 32 % after 24 h (Fig. 6 and Fig. S5).

## Discussion

Green chemistry approaches are preferred for the synthesis of nanomaterials [5]. Replacement of chemicals in synthesis procedure of therapeutic nanomaterials with eco-friendly biological entities definitely helps in minimizing any harmful impact on human health. Biodegradable polymer PLA has been extensively studied for drug delivery applications [15]. Nowadays lot of attention has been paid towards cellular interactions of therapeutic NPs and their toxicity [25], [26]. Surface coatings of NPs affect their fate inside the cells [27]. There has been no conclusive studies regarding any type of NPs, but generally it has been found that use of surfactants most of the times confer toxicity to NPs [28], especially when other chemicals used in the synthesis procedure are non toxic or least toxic. Surfactants like polyvinyl alcohol, polyethylene glycol and polyvinylpyrrolidone have been used most commonly for PLA NPs synthesis and confer toxicity [16], [18], [19], [28], [29]. Keeping this in view, we have used PEs as surfactant and stabilizer for synthesis of safe and stable PLA NPs. We have used leaf extracts of 5 medicinally important plants like *S. cumini*, *B. variegata*, *C. deodara*, *L. japonica* and *E. sphaericus* for PLA NPs synthesis. These plants have already been tested for metallic NPs synthesis and have shown very good stabilizing action [6], [7], [9], [10]. However, synthesis procedure of metallic and polymeric NPs vary to a great extent [6], [15].

**Figure 7:**
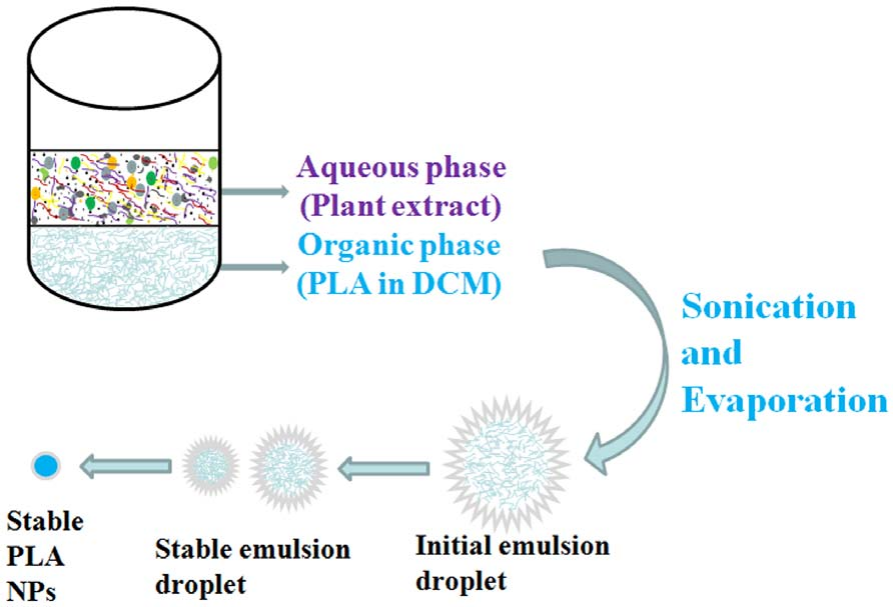
Schematic representation of fabrication of stable PLA NPs by using PEs as stabilizers/emulsifiers.

PEs synthesized PLA NPs were prepared by solvent evaporation method by using PEs as stabilizers/emulsifiers (Fig. 7). In solvent evaporation method PLA polymer was dissolved in organic solvent DCM. The polymer solution was then emulsified with PEs solution. The emulsion droplets were stabilised by the PEs molecules. After the formation of stable emulsion the organic solvent was evaporated by continuous stirring. These droplets were stable during solvent evaporation stage. NPs resulted from single volume shrinkage of the initial emulsion droplet. PEs contain many valuable compounds having properties of stabilizers/emulsifiers [6], [7], [8], [9], [10], [11], [14]. These compounds were observed to act as very good stabilizers for PLA NPs. It has been reported earlier that surfactants play major role in stabilising emulsions formed during NP synthesis [23]. To investigate whether this holds true for PEs as well, a control experiment was performed without PEs solution. Synthesis of PLA NPs was not possible in absence of PEs solution (Fig. S2). Emulsion droplets were not stable and this led to separation of organic and aqueous layers in control experiment (Fig. S2A). This has confirmed that molecules in PEs are acting as stabilizers for emulsions formed during PLA NPs synthesis. Importantly, PEs synthesized PLA-1, PLA-2, PLA-3, PLA-4 and PLA-5 NPs were smaller than <150 nm. On other hand, use of PVA has been reported to synthesize approximately 200 nm sized PLA NPs [23]. Small sized PLA NPs in case of PEs mediated synthesis could be due to presence of biomolecules with different chemical properties. It has been reported earlier that particle size is influenced by the type and concentration of the stabilizers [18]. This holds true in this study as well, where the use of higher concentrations of PEs led to the formation of microparticles. Therefore, PEs mediated synthesis involves a variety of stabilizers and hence the possibility for small size and stabilization.

Morphological characterization by TEM revealed that particles in the size range 70±30 nm to 143±36 nm were formed with these five plants. While DLS characterisation revealed that hydrodynamic diameter of the particles was in the range of 114±70 nm to 164±20 nm. Similar differences in the size of NPs measured by TEM and DLS were also reported in previous study [24]. Polymeric NPs of <100 nm are of special interest for drug delivery applications. The size of nanocarriers as drug delivery vehicle significantly influences the release rate, solubility and dissolution rate of a drug [30], [31], [32]. The size of NPs also has direct relevance with the stability, cellular uptake, bio-distribution and targeted delivery of drugs/small molecules [16], [33], [34], [35]. The polymeric NPs of size <200 nm are ideal for intravenous administration because they can easily pass through the blood capillary [36]. Particle size significantly affects cellular and tissue uptake. Importantly, uptake efficiency of 100 nm size particles has been reported as 2.5–250 folds greater than microparticles [37], [38]. An important criterion for NPs to be introduced in the blood stream is stealth invisibility to the body’s natural defence system. Unless the particles are modelled to escape recognition, mononuclear phagocytic system eliminates them from blood stream [39]. NPs > 200 nm undergo uptake by reticuloendothelial system and are eliminated from the body [15]. Small particles (<100) with a hydrophilic surface have the greatest ability to evade the phagocytic system [40]. Smaller sized NPs (105 nm) are retained in the blood for longer duration as compared to larger NPs (160 nm) [41]. NPs undergo distribution to different organs of the body in a size dependent manner [42]. Clearance organs like kidney and liver can filter particles based on their size. Notably, 100–200 nm NPs showed highest percentage in liver, followed by the kidney and brain [42]. NPs of size < 5.5 nm undergo rapid and efficient urinary excretion and elimination [43]. PEs synthesized PLA NPs were smaller and more uniformly distributed compared to the ones obtained through conventional methods [23], [30], [44]. Small sized PEs synthesized PLA NPs will show lesser uptake by reticuloendothelial system and thus, better cellular uptake and biodistribution. A key goal of PLA NPs is to discharge their payloads specifically at the diseased tissue. Since small sized NPs can have more number of ligands on their surface due to large surface area [40], PEs synthesized PLA NPs may be useful for targeted delivery of small molecules/drugs to diseased tissue.

Zeta potential measurements provide information about the particle surface charge and that determines the performance of the NPs in the body. Also, Zeta potential of NPs is an important criterion for determining their stability against aggregation [45]. Values beyond +/− 30 mV are characteristic for stable colloidal dispersion [46]. The zeta potential evaluation indicated good stability of all PEs synthesized PLA NPs (Fig. 4A–E). All zeta potential values were more than −30 mV showing that they have less tendency for aggregation. Zeta potential values revealed that PEs synthesized PLA NPs were more stable than PLA NPs synthesized by the use of other stabilizers/emulsifiers [47], [48].

Among all PE, *L. japonica* showed the maximum tendency for the formation of PLA NPs (Fig. 1D). Also, *L. japonica* PE synthesized PLA NPs were most uniformly distributed (Fig. 2D) and showed good stability (Fig. 4D). Controlled-release delivery systems deliver small molecules/drugs in the optimum dosage for long periods, thus increasing the efficacy of the drug, maximizing patient compliance and enhancing the ability to use poorly soluble or relatively unstable small molecules/drugs [15]. To check the efficiency of these PEs synthesized PLA NPs for the delivery of small molecules including drug molecules, PLA-4 NPs were selected. PLA-4 NPs have size near 100 nm and strong negative zeta potential. As a matter of proof of concept, we have tested the feasibility of encapsulation and release of a lipophilic molecule quercetin on PE synthesized PLA-4 NPs. This molecule has been reported for antioxidant, anti-cancer and antiviral activities [49], [50], [51]. In spite of proven pharmacological properties, the use of quercetin in pharmaceutical field is limited due to its poor bioavailability, poor permeability, instability and extensive first pass metabolism before reaching the systemic circulation [22].

Quercetin was encapsulated on PE synthesized PLA-4 NPs with maximum encapsulation efficiency (100%). This could be due to the method used for the encapsulation of quercetin. In our previous work where we used PLA NPs synthesized through chemical route, quercetin encapsulation efficiency was 96.7 % [23].

The *in vitro* release studies are important to know the adsorption or encapsulation of quercetin on the PE synthesized PLA-4 NPs. The release rate of quercetin was burst release (up to within initial hours), after which sustained release of the entrapped quercetin was observed. This quercetin loaded PE synthesized PLA-4 NPs has relatively less burst effect and more sustained release characteristics than PLA NPs synthesized by other methods [23]. Rapid initial release was normally attributed to the fraction of quercetin which was adsorbed on the surface of the NPs. Upon addition of the NPs to the release medium, this fraction of quercetin diffused rapidly into the surrounding medium. At the second stage, the release of quercetin was slower and sustained which may be attributed to diffusion of the quercetin entrapped within the core of the NPs.

PEs synthesized PLA NPs showed high retention for quercetin as compared to PLA NPs synthesized using PVA. Quercetin release from PLA NPs is described to be diffusion mediated in the early stages followed by diffusion-erosion mediated in the later stage [23]. Though biomolecules of PEs were not found to be affecting the behaviour of quercetin present on the surface of NPs but they possibly affected the diffusion of quercetin and erosion of polymer matrix. Similar high retention and trend of release has been reported for protein from PLGA NPs [18].

High encapsulation efficiency and slow release makes quercetin loaded PE synthesized PLA-4 NPs a suitable candidate for the further development of nanomedicines. These PE synthesized PLA NPs can also be useful for slow and sustained release of other bioactives like nutraceuticals, flavours and small molecules with better cellular uptake and suitable persistence in the blood stream.

In conclusion, we reported here first time the fabrication of PEs mediated biogenic PLA NPs. Small sized, uniformly distributed and stable NPs in the size range of 70±30 nm to 143±36 nm were produced. As an example to show the use of such PE synthesized PLA NPs, quercetin molecule was loaded successfully on PLA-4 NPs. *In vitro* release study revealed slow and sustained release of quercetin. Therefore, such PLA NPs appeared to be promising candidates for successful development of drug/small molecules delivery vehicles and other applications in which products come in direct human contact.

## Materials and Methods

### Materials

Poly-D,L-lactide (PLA, MW:75,000–120,000), quercetin, HPLC grade acetonitrile (ACN), water, ethanol and trifluoroacetic acid (TFA) were purchased from Sigma-Aldrich. Dichloromethane (DCM) was purchased from Merck. Solutions were prepared using water filtered through a Milli-Q water system (Millipore, Bedford, MA). Plant (*Syzygium cumini*, *Bauhinia variegata*, *Cedrus deodara*, *Lonicera japonica* and *Eleaocarpus sphaericus*) leaves were collected from the Institute CSIR-IHBT campus, Palampur.

### Methods

#### Preparation of plant extracts

Plant extracts (PEs) were prepared by method reported earlier with minor modifications [8], [11]. Fresh leaves dried at 37°C were ground to make fine powder. Four g dry leaf powder was suspended in 50 ml of deionized Milli-Q water, vortexed and incubated overnight at room temperature. The resulting suspensions were centrifuged at 8000 rpm for 5 min at 4°C and supernatant was filtered through 0.2 µm filter. This filtered solution was termed PE and was used for poly (D,L-lactide) (PLA) NPs synthesis.

#### Synthesizing poly (D,L-lactide) (PLA) nanoparticles using plant extract

Briefly, PLA (12.5 mg) was dissolved in 0.5 ml dichloromethane (DCM) and sonicated (Sonics Vibra cell) at 30 % amplitude for 30 s at room temperature. PE solution (1 ml) was added and again sonicated similarly to form emulsion. The emulsion was diluted by adding double distilled water to make final volume 20 ml. The organic solvent (DCM) was evaporated at 40°C. The NPs were purified/separated by centrifugation at 12,000 rpm for 10 min at 10°C and washed two times with distilled water by centrifugation.

#### Synthesis and encapsulation of quercetin in PLA-4 nanoparticles

PLA (12.5 mg) and quercetin (1.25 mg) was dissolved in 0.5 ml DCM and sonicated (Sonics Vibra cell) at 30 % amplitude for 30 s at room temperature. One ml of *L. japonica* extract (4) was added and again sonicated similarly to form emulsion. The emulsion was diluted by adding double distilled water to make final volume 20 ml. The organic solvent (DCM) was evaporated at 40°C. The NPs were purified/separated by centrifugation at 12,000 rpm for 10 min at 10°C and washed two times with distilled water by centrifugation. Blank PLA NPs were prepared following the same procedure. The final lyophilized product was stored at 4°C until further use. This synthesis procedure was repeated 3–4 times to establish the reproducibility of NPs synthesis.

#### Characterization of plant extract synthesized PLA nanoparticles

UV-Vis spectrophotometer (Nanodrop ND-1000) with path length of 1 mm and 2048 element linear silicon CCD array detector was used for obtaining UV-Vis spectra. 2 µl of PEs stabilized PLA NPs were used for the spectroscopic scan analysis from 220 to 700 nm using appropriate blanks.

PEs synthesized PLA NPs were characterized by scanning electron microscope (S-3400 N, Hitachi, Japan). The water suspended NPs solutions were mounted on an aluminium stub using double sided carbon tape. The solution was slowly evaporated at room temperature. The completely dried sample was coated with gold by sputter coating unit at 10 Pascal vacuum for 10 s (E1010 ion sputter Hitachi, Japan). The images were captured on SEM mode at desired magnification.

The shape and size of NPs was determined by transmission electron microscopy. A drop of PEs stabilized PLA NPs was placed on a copper grid and negatively stained with 2% ammonium molybdate. The images were obtained with a Tecnai, Twin 200 KV (FEI, Netherlands) at a bias voltage of 200 kV. Size and Zeta potential of PEs stabilized PLA NPs was measured on DLS (Zetasizer Nano ZS, Malvern Instruments Ltd.) in disposable cells.

#### Encapsulation efficiency of quercetin loaded plant extract synthesized PLA-4 nanoparticles

Encapsulation efficiency of quercetin in PE synthesized PLA-4 NPs was analyzed using validated HPLC method [23]. The supernatant solution (20 µl) of PE synthesized PLA-4 and quercetin loaded PE synthesized PLA-4 NPs was filtered through 0.22 µm filter. The solution was directly injected on HPLC (Waters coupled with photo diode array detector 2998). The reverse phase C18 column (150 mm×4.6 mm, 5 µm size) was used for HPLC separation. Acetonitrile and water (40∶60) were used as mobile phase with flow rate of 1 ml/min. The detection wavelength was selected as 354 nm. HPLC separation was pre-equilibrated with fresh quercetin for qualitative and quantitative analysis. The calibration curve was drawn by preparing different amount of quercetin (0.0125–0.1 mg/ml) vs. peak area of eluted peak. The linearity range of calibration curve was found to be (0.0125– 0.1 mg/ml), while correlation coefficient was 0.971.

The area of eluted peak of supernatants of quercetin loaded PE synthesized PLA NPs was integrated and used for quercetin quantification. The encapsulation efficiency (EE) and the actual drug loading were calculated using the formula:
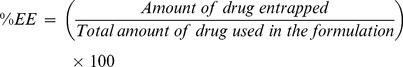
(2)

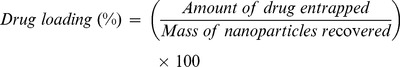
(3)


#### *In vitro* release assay of quercetin loaded plant extract synthesized PLA-4 nanoparticles

Amount of released quercetin was quantified using validated HPLC method with the help of calibration curve. *In vitro* release studies of quercetin loaded PE synthesized PLA NPs were performed by incubating 3.8 mg of quercetin loaded PLA-4 NPs in 10 ml of 0.1 M PBS at pH 7.4. The NPs suspension was continuously stirred in a thermostat (50 rpm) at 37°C. At pre-selected times (0, 0.5, 1, 2, 3, 4, 5 and 24 h), 1.0 ml of sample was taken and lyophilized. This lyophilized solution was dissolved in 100% ACN and quickly centrifuged at 8000 rpm for 5 min to obtain the unreleased quercetin loaded PLA-4 NPs.

The % cumulative release of quercetin was calculated by using following equation:
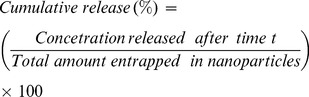
(4)


The released quercetin was subjected for quantitative analysis by validated HPLC method.

## Supporting Information

Figure S1 **Chemical structure of quercetin.**
(TIF)

Figure S2 **Synthesis of PLA NPs in the absence of PE.** Organic and aqueous layers are clearly visible in control experiment (arrow) and no emulsion was formed. While in the left tube clear emulsion can be seen upon addition of PEs during PLA-4 NPs synthesis (A). UV-visible spectra (B) and transmission electron micrograph of control experiment where no PEs was added (C).
(TIF)

Figure S3 **Scanning electron micrographs of PLA NPs synthesized using PEs as stabilizer/emulsifier by solvent evaporation method.** (A) PLA-1 (B) PLA-2 (C) PLA-3 (D) PLA-4 (E) PLA-5.
(TIF)

Figure S4 **HPLC analysis of quercetin loaded PLA-4 NPs.** (A) HPLC chromatograms of standard quercetin (mg/ml). Calibration was obtained by plotting various amounts of quercetin (mg/ml) vs. corresponding HPLC eluted peak area. (B) Chromatogram of quercetin loaded PLA-4 NPs supernatant after separation of synthesized NPs.
(TIF)

Figure S5 **HPLC chromatograms of quercetin released in solutions from quercetin loaded PLA-4 NPs. HPLC chromatograms were obtained for the released quercetin after regular intervals by dissolving in pure acetonitrile.**
(TIF)
