# Supplementary figures and images for: Plant Extract Synthesized PLA Nanoparticles for Controlled and Sustained Release of Quercetin: A Green Approach
Source: PLoS One. 2012 Jul 23;7(7):e41230. doi: 10.1371/journal.pone.0041230 (PMC3402536; doi:10.1371/journal.pone.0041230)

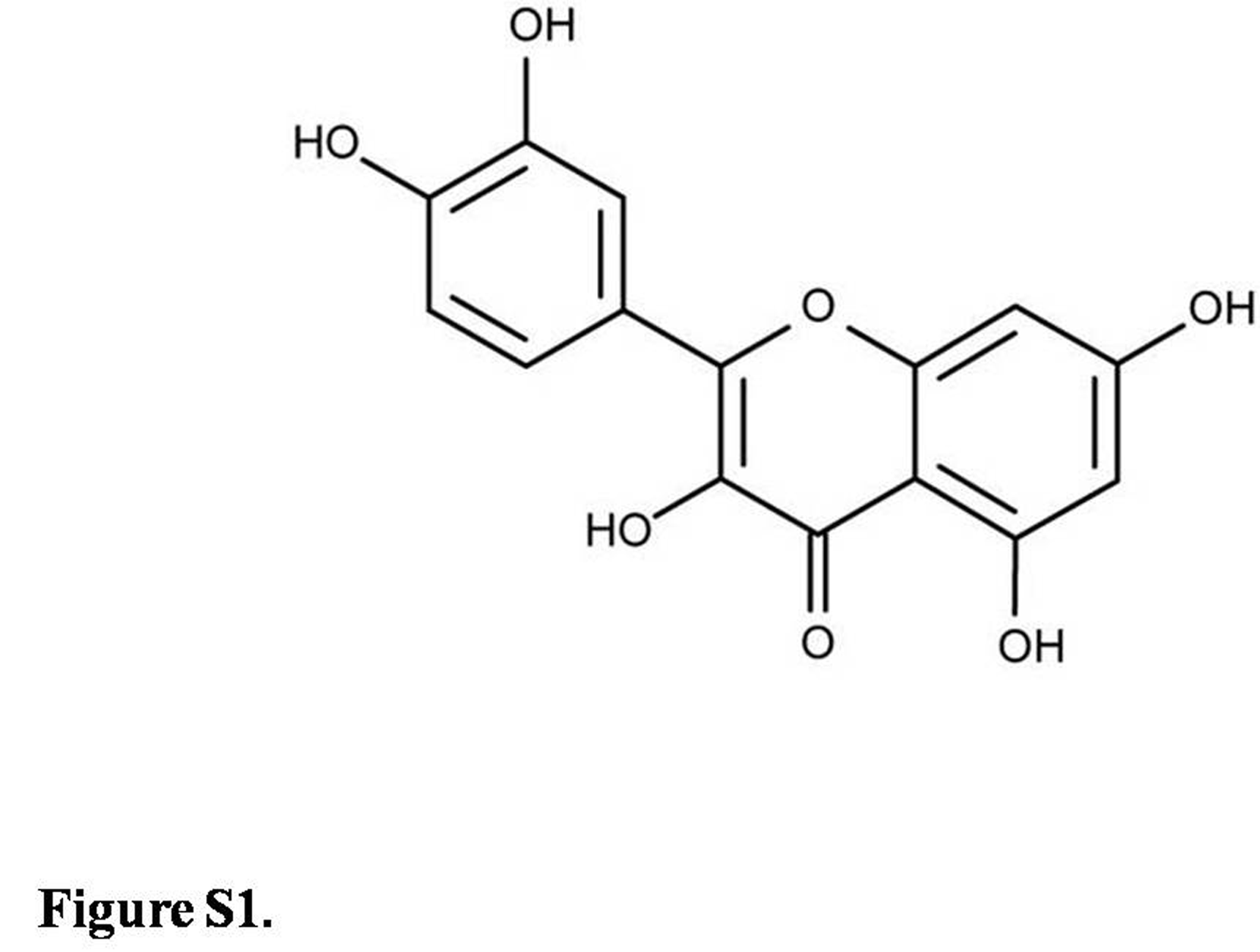

Supplement: Figure S1 — Chemical structure of quercetin. (TIF) [file pone.0041230.s001.tif]

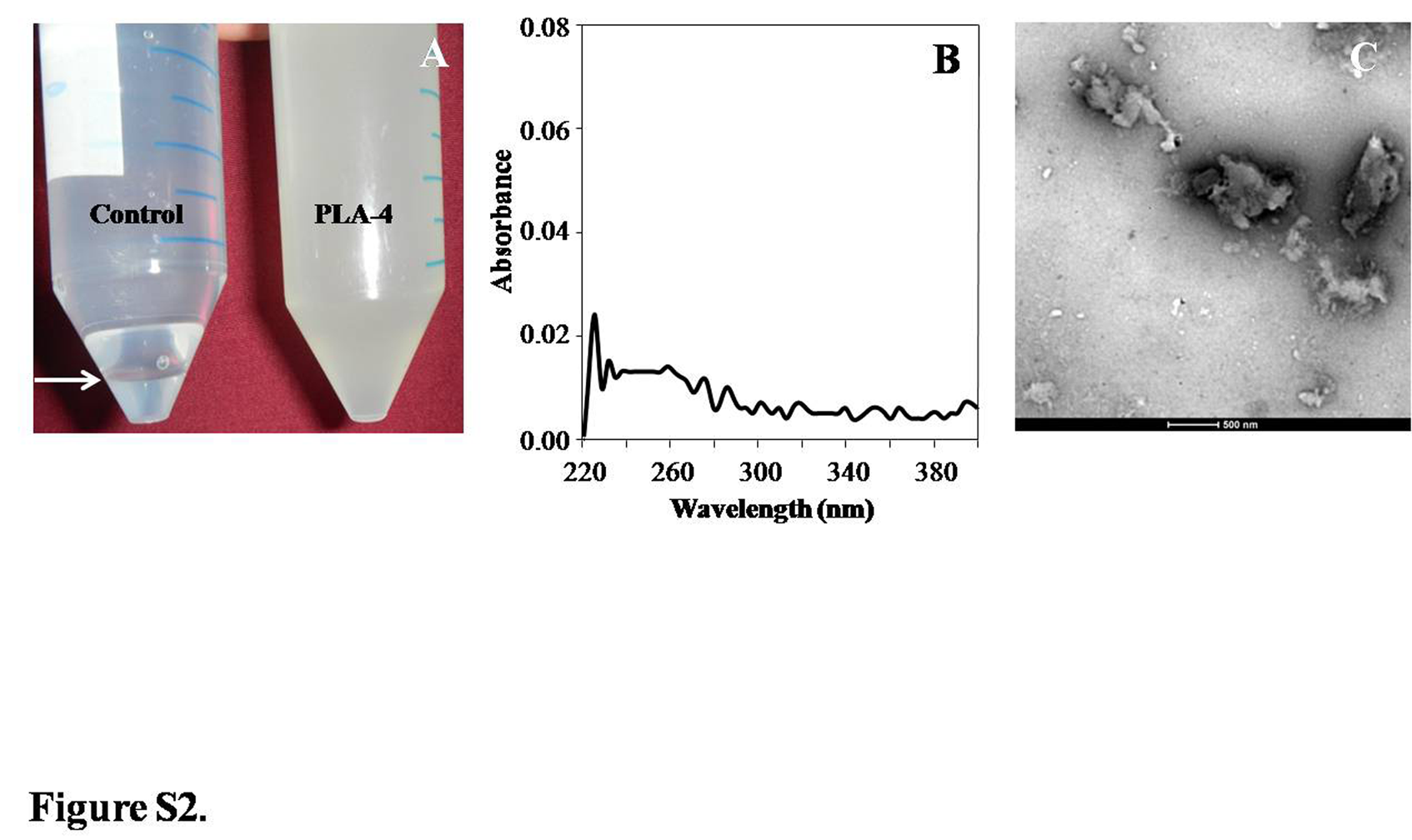

Supplement: Figure S2 — Synthesis of PLA NPs in the absence of PE. Organic and aqueous layers are clearly visible in control experiment (arrow) and no emulsion was formed. While in the left tube clear emulsion can be seen upon addition of PEs during PLA-4 NPs synthesis (A). UV-visible spectra (B) and transmission electron micrograph of control experiment where no PEs was added (C). (TIF) [file pone.0041230.s002.tif]

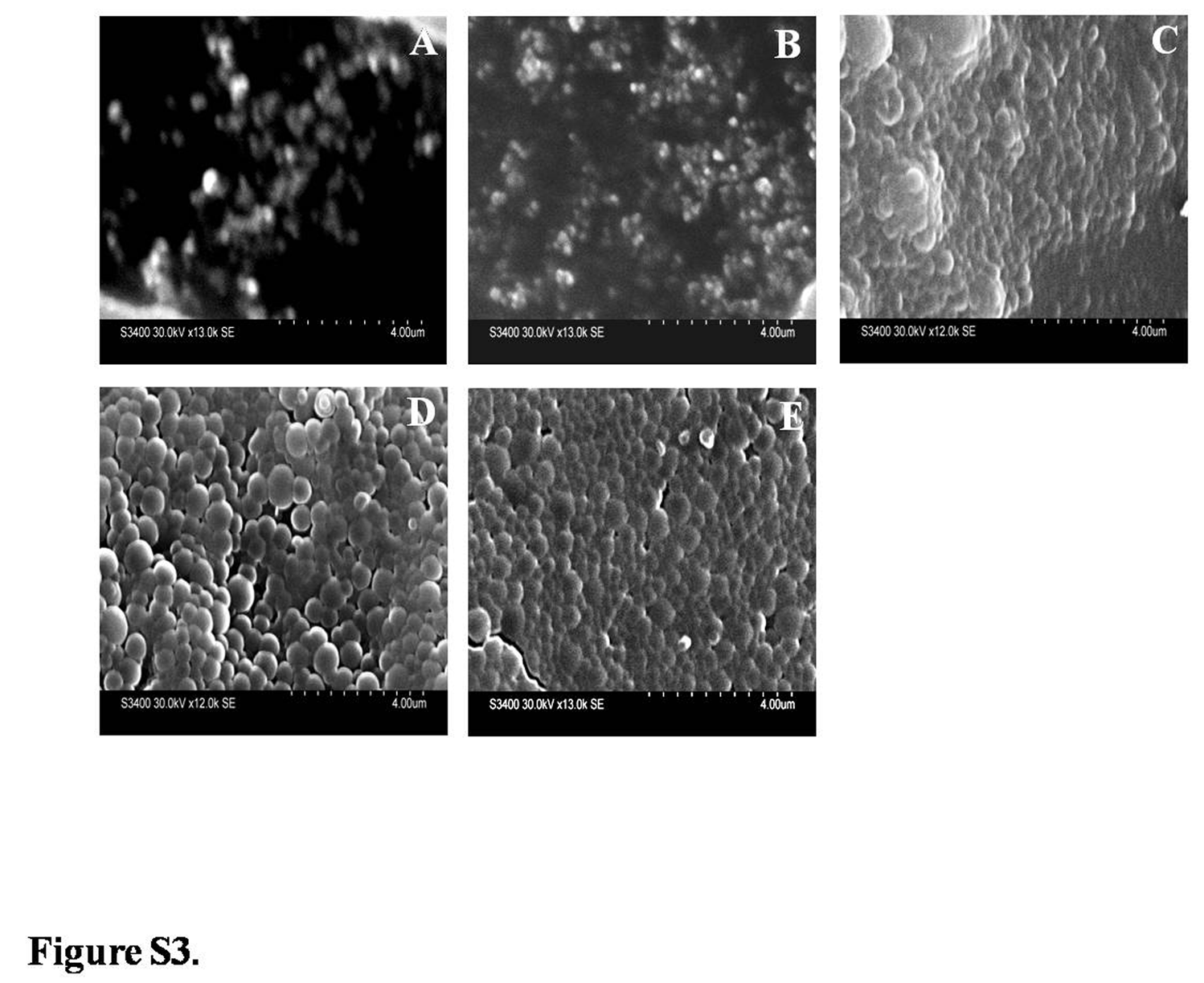

Supplement: Figure S3 — Scanning electron micrographs of PLA NPs synthesized using PEs as stabilizer/emulsifier by solvent evaporation method. (A) PLA-1 (B) PLA-2 (C) PLA-3 (D) PLA-4 (E) PLA-5. (TIF) [file pone.0041230.s003.tif]

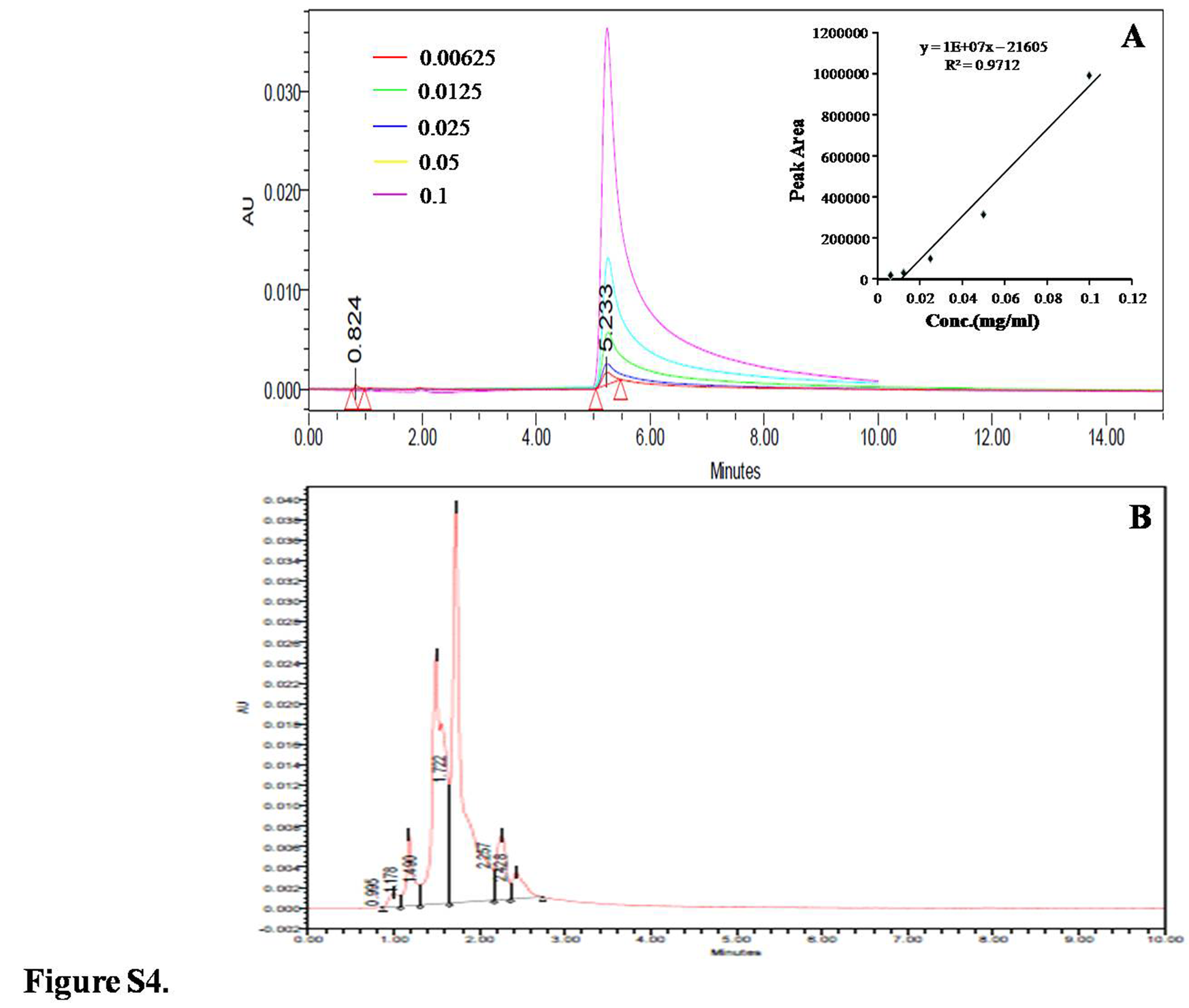

Supplement: Figure S4 — HPLC analysis of quercetin loaded PLA-4 NPs. (A) HPLC chromatograms of standard quercetin (mg/ml). Calibration was obtained by plotting various amounts of quercetin (mg/ml) vs. corresponding HPLC eluted peak area. (B) Chromatogram of quercetin loaded PLA-4 NPs supernatant after separation of synthesized NPs. (TIF) [file pone.0041230.s004.tif]

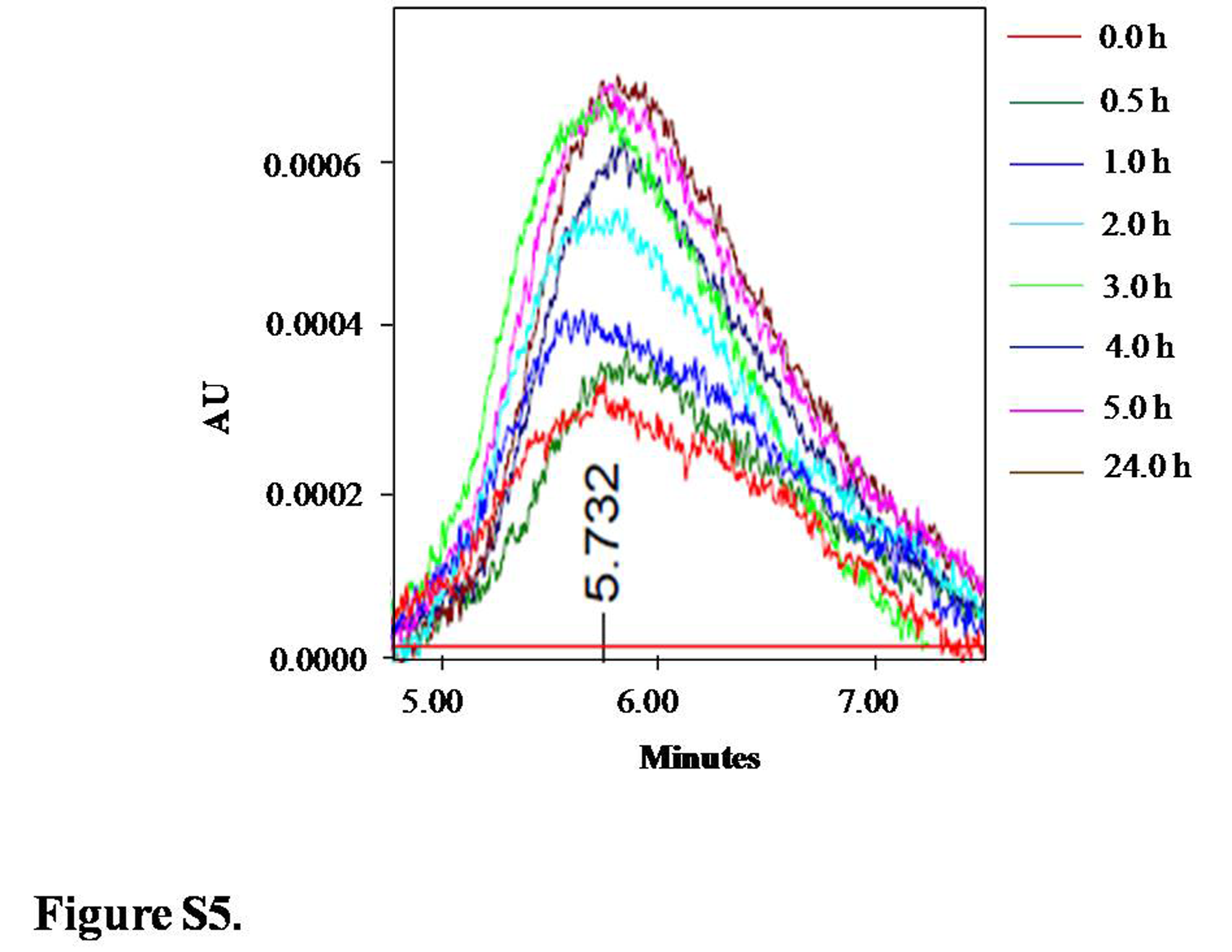

Supplement: Figure S5 — HPLC chromatograms of quercetin released in solutions from quercetin loaded PLA-4 NPs. HPLC chromatograms were obtained for the released quercetin after regular intervals by dissolving in pure acetonitrile. (TIF) [file pone.0041230.s005.tif]
